# The prevention of stroke by statins: A meta-analysis

**DOI:** 10.1097/MD.0000000000030606

**Published:** 2022-09-23

**Authors:** Xiaoxu San, Zhiguo Lv, Peng Xu, Jian Wang, Tianye Lan

**Affiliations:** a School of Traditional Chinese Medicine, Changchun University of Chinese Medicine, Jingyue National High-Tech Industrial Development Zone, Changchun, Jilin, China; b Department of Encephalology, The Affiliated Hospital of Changchun University of Chinese Medicine, Changchun, Jilin, China.

**Keywords:** meta-analysis, randomized controlled trial, statins, stroke

## Abstract

The objectives of this study were to determine the preventive effects of statins on stroke.

**Methods:**

The published randomized controlled trials of statins for stroke prevention were searched from PubMed, EMBASE, Cochrane Library, and China Journal databases. We performed the meta-analysis via calculating the odds ratio (OR) and 95% confidence interval (CI) to study the mortality rate, incidence, and recurrence rate of patients with stroke in the prevention group and the control group. Chi-square-based *Q* test and *I*^2^ statistics were performed to test the potential heterogeneity; we conducted the sensitivity analysis to assess the stability of our analysis. Moreover, we performed the Begg and Egger tests to assess the publication bias.

**Results:**

Nine studies were included to perform meta-analysis, which included 15,497 patients (prevention group [n = 4114]; control group [n = 11383]). We found that the statins were not associated with the patients with stroke in mortality rate (OR = 1.00, 95% CI [0.82, 1.23]) and incidence (OR = 0.94, 95% CI [0.46, 1.92]) between the 2 groups. However, there was a significant differences in recurrence rate between the 2 groups (OR = 0.31, 95% CI [0.19, 0.51]).

**Conclusions:**

Our findings indicated that the statins were associated with the patients with stroke in recurrence rate, but there was no significant correlation with the mortality and morbidity of patients with stroke.

## 1. Introduction

Stroke is a common and serious neurological disease, which can lead to disability and death in severe cases.^[1]^ According to statistics, nearly 20 million people worldwide suffer from stroke each year, with an annual mortality rate of about 25%. The patients with stroke are increasing year by year, and some patients will be permanently disabled due to stroke.^[2]^ Stroke is one of the main reasons for the increasing in global mortality and morbidity. With the gradual severity of the global aging problem, stroke has become a public health problem. Stroke is a disease that is prone to recurrence. Compared to the first stroke, recurrent stroke is more likely to cause disability and death.^[3–5]^ Therefore, the prevention of stroke disease is extremely important.

Statins are 3-hydroxy-3-methylglutaryl coenzyme A reductase inhibitors. Statins could lower blood lipid levels, improve endothelial function, inhibit inflammation, and stabilize atherosclerosis.^[6,7]^ Recent researches have demonstrated that statins could reduce the risk of many cardiovascular diseases, such as ischemic heart disease, hypercholesterolemia, and atherosclerotic cardiovascular diseases, etc.^[8–10]^ Massive studies have shown that statins can improve myocardial infarction and stroke by reducing the levels of serum cholesterol, and the statins can be used in combination with other drugs to treat the patients with stroke.^[11,12]^ However, there are few reports on whether statins can be used to prevent stroke. Thus, the research on the preventive effects of statins on stroke is very important.

In our analysis, the meta-analysis via calculating the odds ratio (OR) and 95% confidence interval (CI) were performed to study the mortality rate, incidence, and recurrence rate of patients with stroke in the prevention group and the control group. This analysis will provide a new understanding for the prevention of stroke.

## 2. Materials and methods

### 2.1. Literature search and selection criteria

A comprehensive literature search of PubMed, EMBASE, Cochrane Library, and China Journal was performed to obtain the randomized controlled trials (RCT) of statins for stroke prevention, and the studies were published before December 2019. The key words included: (“statins” OR “lovastatin” OR “Atorvastatin” OR “cerivastatin”) AND (“Stroke” OR “Fatal stroke” OR “Hemorrhagic stroke”) AND (“Randomized controlled trial” OR “RCT”).

Studies were included if: the research object was a RCT or cohort study of patients with stroke; the prevention group used statins, the control group did not use statins, and the other treatments were the same as those in the prevention group; the outcomes of the study included incidence, mortality, and recurrence rate. Non-authored studies were excluded such as reviews, comments, and conference abstracts.

### 2.2. Data extraction and quality assessment

Eligible research data were independently extracted by 2 researchers into a standardized table, including the first author of the study, year of publication, area of study, treatment plan, sample size, the sex ratio, and age composition of the participants. If there are differences of opinion in the literature data extraction process, then have a group discussion with the third author until a consensus is reached.

We carried out the Cochrane risk assessment tool to evaluate the quality of literature from the following aspects: random sequence generation, allocation concealment, blinding of participants and personnel, blinding of outcome assessment, incomplete outcome data, selective reporting, and other bias.

### 2.3. Statistical analysis

The Review Manager 5.2 was used to conduct meta-analysis. The OR and the corresponding 95% CI were calculated. The Chi-square-based *Q* test and *I*^2^ statistics were used to study the potential heterogeneity among studies. If the heterogeneity test results indicated statistical significance (*P* < .05, *I*^2^ > 50%), we applied the random effect model to merge the data; otherwise, the fixed effect model was applied to merge the data. Moreover, we carried out the Begg and Egger tests to assess the publication bias. All the above *P* values were 2-sided test, and *P* < .05 was considered significant.

## 3. Results

### 3.1. Characteristics of included studies

**Figure 1.**
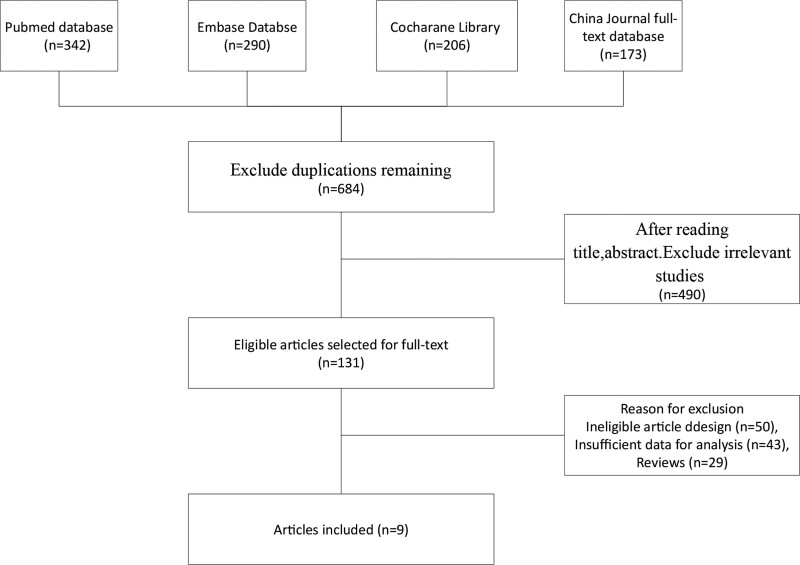
The flow diagram of the study selection.

The study screening steps was displayed in Figure 1. The eligible studies were identified from 4 databases, including PubMed (n = 342), Embase (n = 290), Cochrane library (n = 206), and China Journal (n = 173). Six hundred eighty-four articles remained after excluding duplicate documents, and 490 documents that obviously did not meet the inclusion criteria were eliminated after browsing the titles and abstracts. We obtained 194 studies by titles and abstracts after removing the duplicates. After reviewing the full-text, 50 unqualified articles were eliminated, 43 articles with insufficient analysis data, and 29 reviews were all eliminated. Finally, 9 studies that met the criteria were contained in this meta-analysis.^[13–21]^

As shown in Table 1, a total of 9 RCTs and 15,497 study subjects (prevention group [n = 4114], control group [n = 11383]) were contained in this meta-analysis. The included eligible studies were published from 2002 to 2019, and the locations of studies were mainly in Asia, America, and Europe.

**Table 1:** Characteristics of studies included in the meta-analysis.

Study	Year	Language	Country	No. of patients (male/female)	Age range (mean)	No. of statin group	No. of non-statin group	Years of onset
Dongen	2019	English	The Netherlands	591/344	45 ± 4.4	438	497	January 1994 to May 2007
Garcia	2019	English	Spain	7353/665	61.93 ± 9.97	736	7282	July 2006 to December 2008
Hosomi	2017	English	Japan	1087/491	66.3 ± 8.3	793	785	March 2004 and February 2009
Jin	2019	Chinese	China	103/50	55.9 ± 12.5	114	39	October 2016 to October 2017
Liang	2010	Chinese	China	37/13	64.8 ± 5.2	25	25	May 2003 to January 2008
Liang	2016	Chinese	China	53/25	68 ± 5	39	39	June 2013 to June 2015
Scheitz	2013	English	Germany	781/665	76 ± 7.5	317	1129	February 1998 to December 2012
Waters	2002	English	America	2012/1074	72 ± 12	1538	1548	June 1996 to December 2001
Yum	2017	English	Korea	83/70	68.02 ± 9.86	114	39	April 2003 to June 2014

### 3.2. Meta-analysis

#### 3.2.1. The mortality rate of stroke.

**Figure 2.**
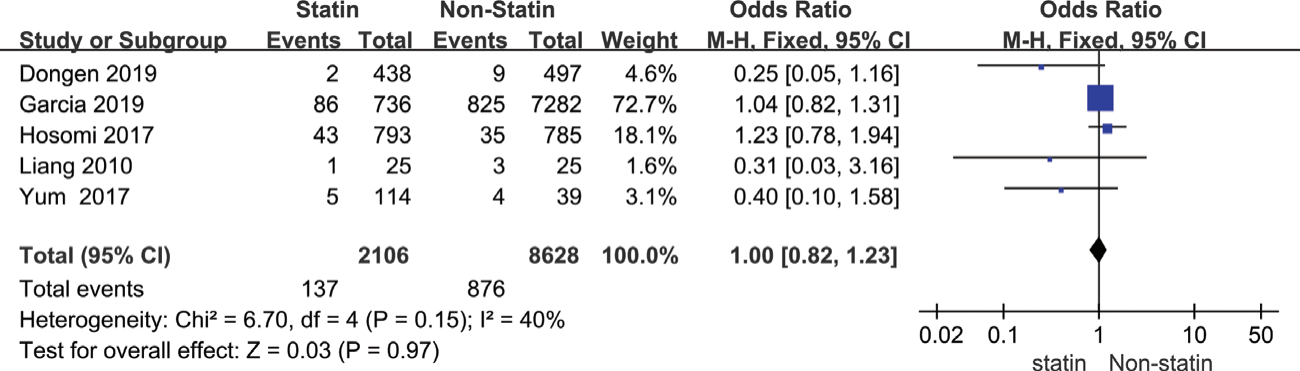
Forest plot of the statin use for the mortality rate of stroke.

The heterogeneity analysis indicated that the model of mortality rate was not statistically different in heterogeneity (*P* = .97, *I*^2^ = 40%), thus we carried out the fixed-effect model to calculate the combined OR value and its 95% CI. We carried out a meta-analysis of the mortality of patients with stroke in the prevention group and the control group after treatment. The results suggested that there was no significant difference in mortality between the 2 groups (OR = 1.00, 95% CI [0.82, 1.23]) (Fig. 2).

#### 3.2.2. The incidence of stroke.

**Figure 3.**
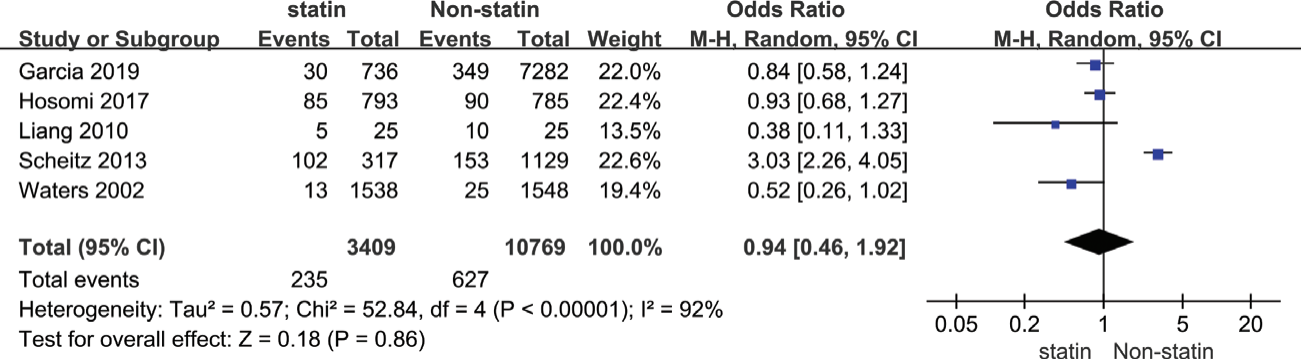
Forest plot of the statin use for the incidence of stroke.

The heterogeneity analysis indicated that the model of incidence was statistically different in heterogeneity (*P* = .86, *I*² = 92%), thus the random effect model was carried out to calculate the combined OR value and its 95% CI. The meta-analysis of the incidence of patients with stroke in the prevention group and the control group was performed, we found that there was no significant difference in incidence between the 2 groups (OR = 0.94, 95% CI [0.46, 1.92]) (Fig. 3).

#### 3.2.3. The recurrence rate of stroke.

**Figure 4.**
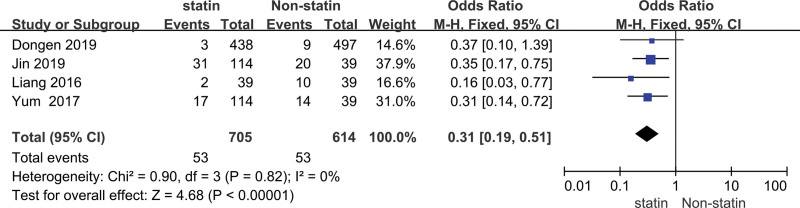
Forest plot of the statin use for the recurrence rate of stroke.

The heterogeneity analysis indicated that the model of recurrence rate was not statistically different in heterogeneity (*P* < .0001, *I*^2 ^= 0%), thus we carried out the fixed-effect model to calculate the combined OR value and its 95% CI. The meta-analysis of the recurrence rate of patients with stroke in the prevention group and the control group was performed, we found that there was a significant difference in the recurrence rate of stroke between the 2 groups (OR = 0.31, 95% CI [0.19, 0.51]) (Fig. 4).

### 3.3. Sensitivity analysis

**Figure 5.**
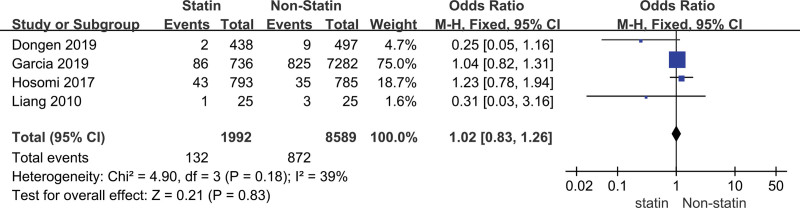
Forest plot of the sensitivity analysis of this study.

The sensitivity of the comprehensive analysis results was examined by deleting the study and integrating the results of the remaining studies. The sensitivity analysis was carried out by removing Yum2017 articles, and the results showed that the change was small, ranging from 40% to 39% (Fig. 5), which indicated that the results we included in the article were robust.

### 3.4. Publication biases

**Figure 6.**
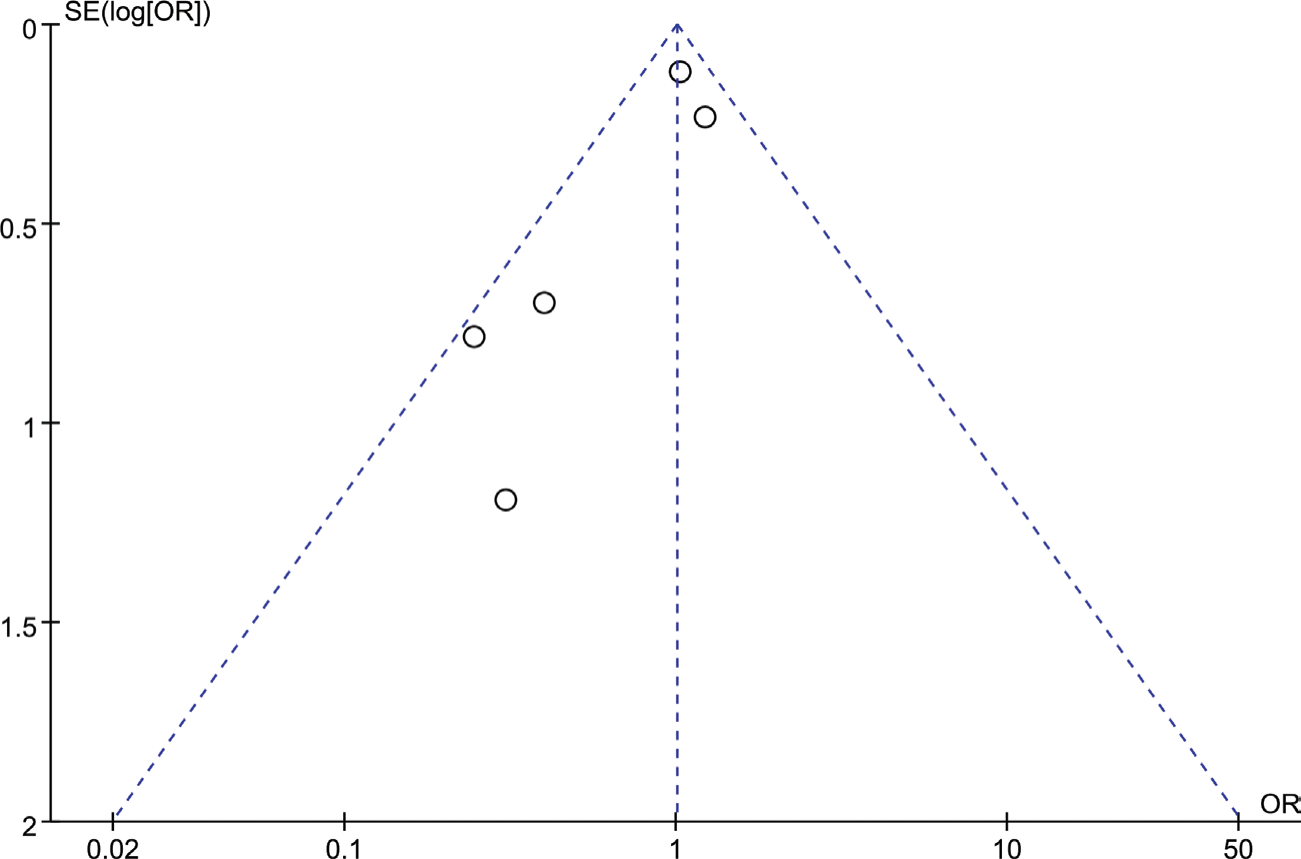
Funnel plot of the publication bias analysis of this study.

**Figure 7.**
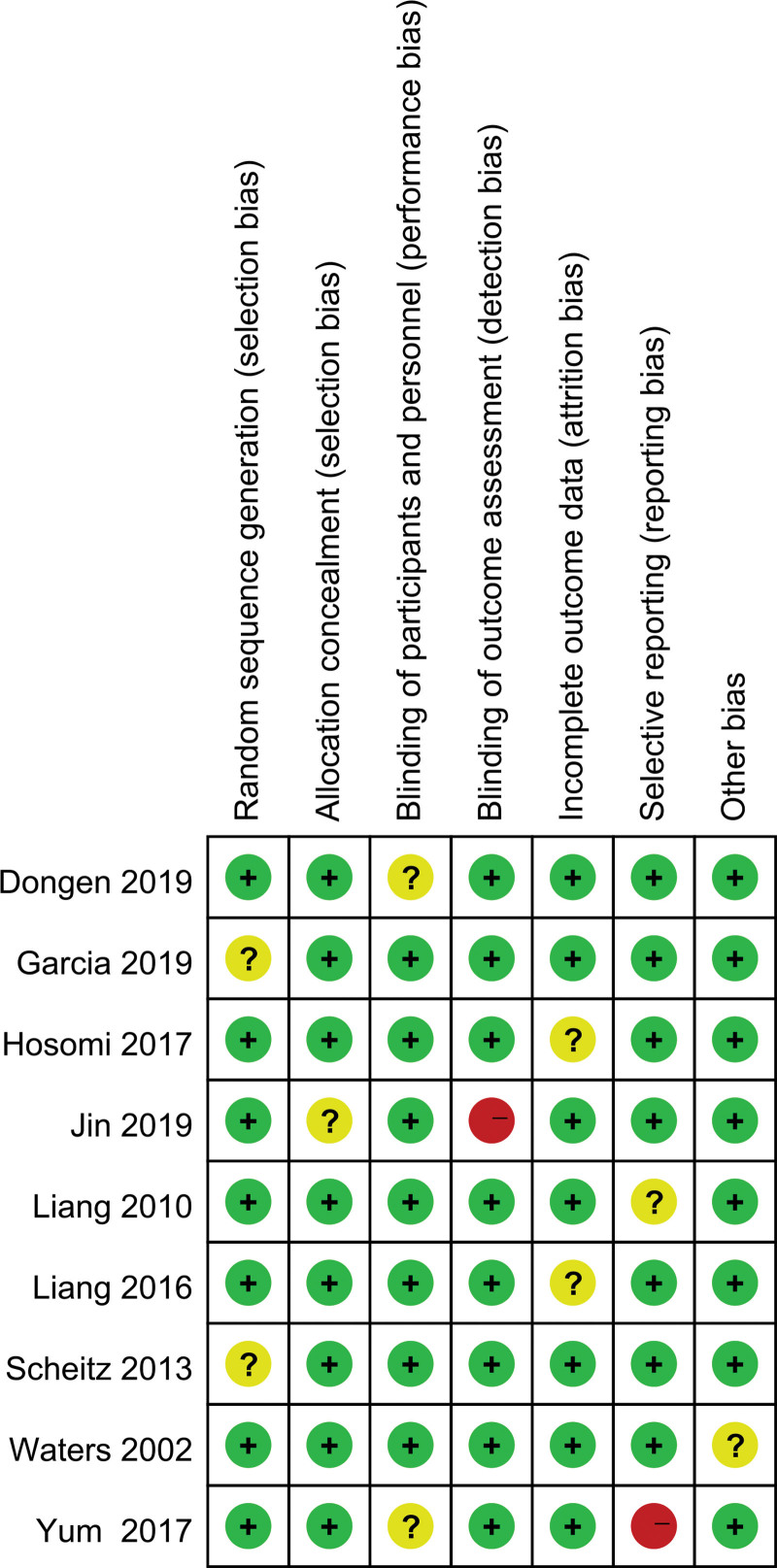
Risk evaluation of bias in included studies. Red, yellow, and green indicated high risk, unclear risk, and low risk of bias, respectively.

The potential publication biases test was performed on all models of this study. We found that the research points were basically symmetrically distributed on both sides of the axis, and there was no serious publication bias, so the conclusions drawn were relatively reliable (Fig. 6). Moreover, we found that none of the included 9 trials had high-risk biases (Fig. 7).

## 4. Discussion

Stroke is a common cerebrovascular disease.^[22]^ In recent years, many researches have reported that in the prevention of massive cerebrovascular disease, the application of statins for lipid-lowering therapy can significantly reduce the occurrence of cerebrovascular events.^[23–25]^ Therefore, statins have also begun to enter the prevention research of ischemic stroke. It is well known that the main effect of statins is to lower blood lipids. Moreover, recent clinical trial evidences have showed that statins have anti-inflammatory effects, which plays a vital role for the prognosis and treatment of cardiovascular diseases.^[26,27]^ At present, lots of researches have reported that statins can be used to treat stroke. For example, Babak et al found that statins could reduce the concentration of C reaction protein in patients with stroke and exert a certain anti-inflammatory effect,^[28]^ which indicated that statins could be used as potential anti-inflammatory drugs for patients with stroke. Rodriguez et al identified that statins could treat atherosclerotic cardiovascular disease by lowering the level of low-density lipoprotein cholesterol.^[29]^ Waters et al confirmed that patients with acute coronary syndrome were treated with atorvastatin, which reduced the incidence of stroke by lowering the cholesterol level.^[13]^ However, the preventive effect of statins on stroke is still uncertain.

In our meta-analysis, the association between the statins and the prevention of stroke was evaluate. Our results indicated that the statins were not associated with the patients with stroke in mortality rate and incidence. Another finding we found is that there was a significant difference in the recurrence rate of stroke. Our results indicated that statins have no definite preventive effect on stroke. Zhang et al found that the combination use of atorvastatin and other drugs in a rat model can reduce the incidence of hemorrhagic stroke.^[30]^ Lu et al demonstrated that the combination use of rosuvastatin and other drugs after a stroke can prevent the stroke from turning into the hemorrhagic stroke.^[31]^ Our results suggested that statins may need to be used in combination with other drugs to prevent stroke.

Several sources of heterogeneity need to be explained. In our comprehensive analysis, the heterogeneity test results are partly significant, and the reason may be differences caused by different regions, such as differences in regional living habits, living environment, and economic development level. In addition, the influence of confounding factors such as gender and age may also bring about heterogeneity.

Several limitations exist in our analysis. First, the final selected studies and the participated patients were limited. Second, we cannot include individual patient-level data, only data from research groups based on published studies were included. This is also an inherent limitation of many meta-analyses. There was the lack of clinical data on randomized clinical trials in stroke populations and clinical data on the use of statins in young populations. Finally, heterogeneity is also one of the limitations of this analysis.

Our analysis also has several advantages. Our analysis included RCT, the baseline homogeneity of the prevention group and the control group was good, and the reliability of the results was high.

## 5. Conclusion

In summary, the association between statins and stroke was evaluated. Our analysis showed that the statins were associated with the patients with stroke in recurrence rate, but there was no significant correlation with the mortality and morbidity of patients with stroke. These findings may indicate that statins could reduce the risk of recurrence in patients with stroke but have no obvious preventive effect on stroke.

## Author contributions

**Conceptualization:** Xiaoxu San.

Data curation: Jian Wang.

Formal analysis: Zhiguo Lv, Peng Xu.

Methodology: Tianye Lan.

Writing – original draft: Xiaoxu San, Zhiguo Lv.

Writing – review & editing: Xiaoxu San, Zhiguo Lv, Peng Xu, Jian Wang, Tianye Lan.

## Correction

Tianye Lan was removed as one of the corresponding authors. Jian Wang’s email address was updated to jian-w222@163.com.
